# Interaction of serum amyloid P component with hexanoyl bis(d-proline) (CPHPC)

**DOI:** 10.1107/S1399004714013455

**Published:** 2014-07-25

**Authors:** Simon E. Kolstoe, Michelle C. Jenvey, Alan Purvis, Mark E. Light, Darren Thompson, Peter Hughes, Mark B. Pepys, Stephen P. Wood

**Affiliations:** a Laboratory of Protein Crystallography, Wolfson Drug Discovery Unit, Centre for Amyloidosis and Acute Phase Proteins, Division of Medicine (Royal Free Campus), University College London, Rowland Hill Street, London NW3 2PF, England; b School of Biological Sciences, University of Southampton, Southampton SO17 1BJ, England; c Department of Life Sciences, Sir Ernst Chain Building, Imperial College London, London SW7 2AZ, England; d UK National Crystallography Service, School of Chemistry, University of Southampton, Southampton SO17 1BJ, England; e School of Life Sciences, University of Sussex, Falmer, Brighton BN1 9RQ, England

**Keywords:** serum amyloid P component, CPHPC, amyloidosis

## Abstract

Under physiological conditions, the pentameric human plasma protein serum amyloid P component (SAP) binds hexanoyl bis(d-proline) (*R*-1-{6-[*R*-2-carboxy-pyrrolidin-1-yl]-6-oxo-hexanoyl}pyrrolidine-2-carboxylic acid; CPHPC) through its d-proline head groups in a calcium-dependent interaction. Cooperative effects in binding lead to a substantial enhancement of affinity. Five molecules of the bivalent ligand cross-link and stabilize pairs of SAP molecules, forming a decameric complex that is rapidly cleared from the circulation by the liver. Here, it is reported that X-ray analysis of the SAP complex with CPHPC and cadmium ions provides higher resolution detail of the interaction than is observed with calcium ions. Conformational isomers of CPHPC observed in solution by HPLC and by X-ray analysis are compared with the protein-bound form. These are discussed in relation to the development of CPHPC to provide SAP depletion for the treatment of amyloidosis and other indications.

## Abstract 2

Serum amyloid P component is a pentameric plasma glycoprotein that recognizes and binds to amyloid fibres in a calcium-dependent fashion and is likely to contribute to their deposition and persistence *in vivo*. Five molecules of the drug CPHPC avidly cross-link pairs of protein pentamers and the decameric complex is rapidly cleared *in vivo*. Crystal structures of the protein in complex with a bivalent drug and cadmium ions, which improve crystal quality, allow the definition of the preferred bound drug isomers.

## 1. Introduction   

Serum amyloid P component (SAP) is a normal plasma glycoprotein that recognizes and binds to amyloid fibres in a calcium-dependent fashion wherever these are deposited in tissues (Pepys, 2006 ▶). Radiolabelled SAP is a scintigraphic tracer that is used to image amyloid in the diagnosis and management of amyloidosis (Hawkins & Pepys, 1995 ▶). SAP stabilizes amyloid fibres *in vitro* and is likely to contribute to their deposition and persistence *in vivo*, validating the interaction as a therapeutic target in amyloidosis (Tennent *et al.*, 1995 ▶). The common motif that enables recognition by SAP of fibres produced by a range of different proteins is not known, but the interaction with *O*-phosphothreonine may explain how SAP binds to neurofibrillary tangles of tau protein (Kolstoe *et al.*, 2009 ▶). The first small-molecule ligand shown to interact with SAP in a calcium-dependent reaction that competed with fibre recognition was the pyruvate acetal of galactose, methyl 4,6-*O*-(1-carboxyethylidene)-β-d-galactopyranoside (MOβDG), a trace constituent of agarose and a cell-wall component of some bacteria (Hind *et al.*, 1984 ▶). This ligand apparently blocks SAP binding to amyloid fibres by occupying the pocket formed by calcium-ligating loops located on each subunit, identifying this as the most likely amyloid-recognition region. Two calcium ions are bound about 4 Å apart on each SAP subunit and are stabilized by interaction with a range of carboxylate and amide side chains. This site is replicated five times on one face, the binding (B) face of the SAP pentamer, suggesting that fibre-binding affinity may be amplified by a multi-point attachment process. The high-affinity ligand CPHPC was later produced in a medicinal chemistry programme following an initial high-throughput screen for inhibitors of the binding of human SAP to amyloid fibrils formed from the Alzheimer’s disease Aβ_1–42_ peptide (Pepys *et al.*, 2002 ▶). Although each d-proline head group, represented by free *N*-acetyl-d-proline, is weakly bound (*K*_d_ = ∼15 µ*M*), the bivalent bis(d-proline) derivative is avidly bound with a *K*_d_ of ∼10 n*M*, reflecting the multivalency gain of avidity achieved when five ligand molecules cross-link two pentameric SAP molecules (Pepys *et al.*, 2002 ▶). *In vivo*, these cross-linked complexes are rapidly cleared from the circulation by the hepatocytes, which is a novel pharmacological mechanism for depleting a specific plasma protein.

Structural studies of the SAP–CPHPC complex by X-ray diffraction at medium resolution (3.2 Å) showed that in addition to a carboxylate interaction with the calcium site of SAP, the pyrrolidine ring of the d-proline constituents of CPHPC bound into a hydrophobic pocket formed by the side chains of Tyr74, Tyr64 and Leu62. The approach of each d-proline head group to the opposing sites of the in-register twofold axis-related pairs of subunits of the SAP decamer appeared to be facilitated by a kinked rotamer at the centre of the alkyl linker (Pepys *et al.*, 2002 ▶). This study was not able to clearly define the geometry of the peptide bond preceding the proline as *cis* or *trans*. Subsequent studies employing an alkyl or a piperazine linker to couple cyclic pyruvate ketals of glycerol as head groups allowed a substantial rotation between opposing pentamers (more than 20°) of the stabilized decamer (Ho *et al.*, 2005 ▶).

In this paper, we revisit the details of the structural analysis of calcium-bound SAP in complex with CPHPC and present a higher resolution study enabled by crystallization of the complex with cadmium ions in order to provide guidance for the design of a more rigid ligand. We also report a high-resolution analysis of the interaction of *N*-acetyl-d-proline, a substructure of CPHPC, with SAP in comparison with the l-isomer. These crystallographic investigations are complemented by calorimetric studies of ligand affinity and measurements of the conformational flexibility of CPHPC in solution, providing a number of parallels with earlier investigations of the angiotensin-converting enzyme inhibitor captopril (Nishikawa *et al.*, 2004 ▶).

## 2. Materials and methods   

### 2.1. Protein isolation   

SAP was purified as described previously (Hawkins *et al.*, 1991 ▶; Pontet *et al.*, 1978 ▶). Briefly, the protein was captured from serum by calcium-dependent binding to phosphoethanolamine covalently immobilized on carboxyhexyl Sepharose. C-reactive protein, which also binds to phosphoethanolamine, was removed by elution with phosphocholine, and the SAP was then eluted by calcium chelation with EDTA prior to gel filtration and concentration by ultrafiltration. The protein preparation carried forward for crystallization contained no calcium ions.

### 2.2. Co-crystallizations   

*N*-Acetyl-l-proline was obtained from Sigma–Aldrich, *N*-acetyl-d-proline was obtained from Bachem AG and CPHPC was synthesized as described previously (Pepys *et al.*, 2002 ▶). SAP at 11 mg ml^−1^ was incubated with various molar excesses of ligands at room temperature in 10 m*M* Tris–HCl buffer pH 8.0, 140 m*M* NaCl, 5 m*M* CaCl_2_ or cadmium acetate for approximately 2 h before being diluted with well solution in a 1:1 ratio. Hanging-drop vapour-diffusion screens were constructed from previously known SAP crystallization conditions and using Molecular Dimensions Structure Screens.

### 2.3. Crystal structure determination of SAP–ligand complexes   

All crystals were vitrified at 100 K with 20%(*v*/*v*) glycerol as a cryoprotectant. X-ray diffraction data were collected on stations I03 at Diamond Light Source (DLS), Oxfordshire, England and ID14-1 and ID14-2 at the ESRF, Grenoble, France and were processed using *MOSFLM* (Leslie, 2006 ▶), *XDS* and programs from the *CCP*4 suite (Winn *et al.*, 2011 ▶) or *DENZO*/*SCALEPACK* (Otwinowski & Minor, 1997 ▶). *MOLREP* (Vagin & Teplyakov, 2010 ▶) or *Phaser* (McCoy *et al.*, 2007 ▶) and PDB entry 1sac (Emsley *et al.*, 1994 ▶) were used for molecular replacement. The structures were initially refined with *SHELXL* (Sheldrick, 2008 ▶), *CNS* (Brünger *et al.*, 1998 ▶) or *REFMAC* (Murshudov *et al.*, 2011 ▶), but were all completed with *PHENIX* (Adams *et al.*, 2010 ▶); models were built with *Coot* (Emsley & Cowtan, 2004 ▶). Validation was performed with *MolProbity* (Chen *et al.*, 2010 ▶). Completed structures were deposited in the PDB as entries 4avs, 4ayu, 4avt and 4avv.

### 2.4. Crystal structure determination for CPHPC   

CPHPC was crystallized in batch from cooling a 100 mg ml^−1^ solution in aqueous acetone [80:20(*v*:*v*)] from 50°C to room temperature. Data were collected by the EPSRC small-molecule National Crystallography Service at the University of Southampton. Intensities were recorded at 120 K using a Bruker Nonius Kappa CCD Roper area-detector diffractometer mounted at the window of a rotating Mo-anode (Mo *K*α, 0.71073 Å) generator. Unit-cell determination, data collection and processing were carried out using *DirAx* (Duisenberg, 1992 ▶), *Collect* (Nonius) and *DENZO* (Otwinowski & Minor, 1997 ▶) and a multi-scan absorption correction was applied using *SADABS* (Sheldrick, 1997*a* ▶). The structure was solved by direct methods and was refined by full-matrix least squares (Sheldrick, 1997*b* ▶) on *F* ^2^ (Friedel opposites were merged). With the exception of those on the water molecule (freely refined), all H atoms were placed in idealized positions and were refined using a riding model. The five- membered ring was modelled as disordered between two very close possible conformations. Thermal parameter constraints were applied to equivalent atoms across the two parts. Occupancy values were initially refined and were then fixed in the final refinement cycles.

### 2.5. HPLC of CPHPC   

CPHPC dissolved at 10 mg ml^−1^ in aqueous mobile phase was applied onto a 4.6 × 150 mm Gemini 3 µm C6-Phenyl reversed-phase column (Phenomenex) equilibrated with 100 m*M* ammonium acetate at pH 4.5 or 7.5 and eluted at 1 ml min^−1^ with an acetonitrile gradient using a Gilson chromatograph. UV absorption was monitored at 220 nm. Samples were cooled to 4°C before application onto the column and were eluted at this temperature in an attempt to trap the isomer composition.

### 2.6. Calorimetry   

Isothermal titration calorimetry was performed using a MicroCal VP-ITC (MicroCal Inc., Northampton, Massachusetts, USA). Ligand was titrated into protein solution at molar ratios of between 20:1 and 25:1, corresponding to approximately 2 m*M* ligand and 80 µ*M* protein, in a buffer consisting of 200 m*M* CaCl_2_, 10 m*M* Tris–HCl pH 8.0, 140 m*M* NaCl, 0.1%(*w*/*v*) NaN_3_. A high calcium chloride concentration was used to prevent the calcium-dependent auto-aggregation of isolated human SAP which occurs at physiological ionic strength (Hutchinson *et al.*, 2000 ▶). Results were averaged over three experiments and *K*_d_ was estimated from curve-fitting enthalpy changes using the *Origin*7 program.

## 3. Results   

### 3.1. The SAP–CPHPC complex structure   

**Figure 1:**
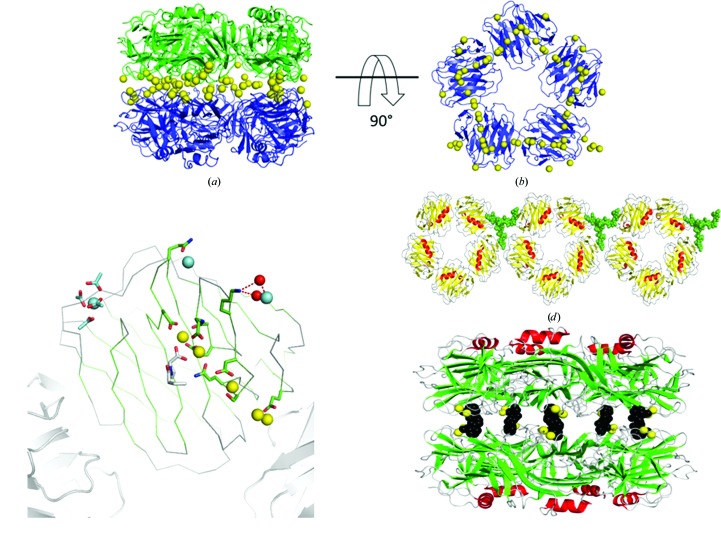
(*a*) The CPHPC-bound decamer of SAP viewed normal to the fivefold axis and showing the 70 cadmium ions bound (yellow spheres). (*b*) Removing one pentamer and viewing down the fivefold axis shows a pentagonal string of metal ions trapped within the decamer and peripheral sites. (*c*) C^α^ trace of subunit *A* with eight cadmium ions, five of which (yellow spheres) form an extended line and become buried in the decamer and three of which (cyan spheres) bind at the periphery. Portions of two adjacent subunits of the pentamer are shown as grey strands. CPHPC is shown with grey C atoms, subunit side chains with green C atoms, acetates in cyan and waters as red spheres. (*d*) The tentative positioning of one partially ordered sugar chain (green spheres) on the SAP pentamer (yellow strands, red helices). (*e*) Decamer of SAP with five CPHPC molecules (black) cross-linking pentamers (green strands and red helices) between their metal sites (yellow).

Crystals of the SAP–CPHPC complex grown in the presence of calcium ions and a tenfold molar excess of CPHPC at 4°C have been reported previously (Pepys *et al.*, 2002 ▶). The crystals contained a large tetragonal unit cell containing approximately 83% solvent and ten SAP sub­units in the asymmetric unit. The crystals did not tolerate removal from the cold room prior to vitrification, and not unexpectedly their diffraction was relatively weak (limited to 3.2 Å resolution). Radiation damage caused rupture of some disulfide bridges, but the conformational effects were local. This structure was determined using molecular replacement, and here we have revisited the refinement in the light of developments in procedures for medium-resolution data. Statistics are included in Table 1 ▶. A new crystal form of this complex was produced from a variant of Molecular Dimensions Structure Screen II condition No. 38 consisting of 100 m*M* cadmium chloride, 100 m*M* sodium acetate buffer pH 4.6, 30%(*v*/*v*) PEG 400. The monoclinic crystals had a much lower solvent content (63%) and data were collected on BM14-1 at the ESRF to 1.6 Å resolution (see Table 1 ▶). A molecular-replacement solution was found using *MOLREP* with a single SAP pentamer (PDB entry 1sac) as the search model with all ligands and Ca atoms removed. Analysis of the resulting maps indicated prominent difference electron density for multiple divalent cations bound to each SAP subunit. Two were located within the ligand-binding site of each SAP subunit, where calcium ions are known to bind in other structures of SAP, and three more were bound per subunit at the interface between pentamers, ligated by Glu14 and Asp145. The strong electron density, the mother-liquor composition and the coordination partners suggest that these are cadmium ions. There are 35 cadmium ions in the asymmetric unit: 25 are bound at the interface between pentamers and a further ten cadmium ions are bound at the periphery and form lattice contacts (see Figs. 1 ▶ *a*, 1 ▶ *b* and 1 ▶ *c*). Overall, the decamer of SAP contains 50 cadmiums at the interface and 20 more at the surface. Several of the cadmium ions are encircled by electron-density features that we have built as acetate ions from the buffer. Approximately 1000 water molecules were added to the structure to give a final *R* factor of 15.0% and a final *R*_free_ of 17.5%. The overall structure of the protein component of these complexes is very similar to the previously reported structure.

**Figure 2:**
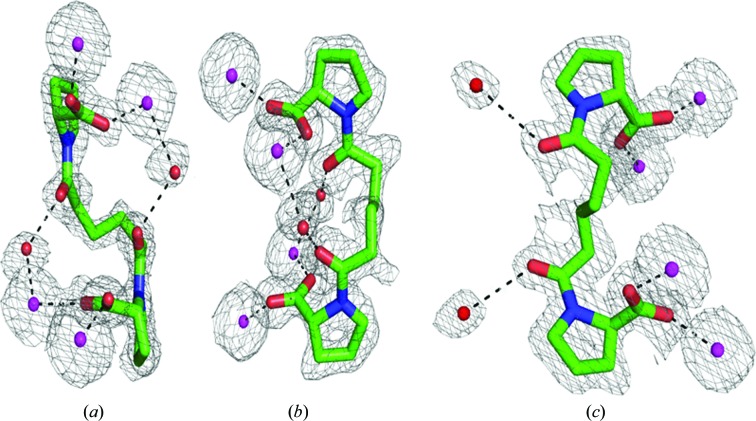
Conformation of CPHPC in bound *trans* (*a*, *b*) and *cis* (*c*) forms together with 2*mF*_o_ − *DF*_c_ OMIT maps contoured at 1.2σ and carved at 1.6 Å around ligand atoms in *PyMOL* (DeLano, 2002 ▶). Orthogonal views of the same site in (*a*) and (*b*) show metal and water ligation and the kinked alkyl chain, while views (*b*) and (*c*) show the distinct differences in the interactions of the *trans* and *cis* isomers with water molecules. Cadmium ions and water molecules are shown in magenta and as red spheres, respectively; CPHPC C atoms are in green.

In summary, the structure of SAP is comprised of five identical 204-residue subunits associated in a ring 95 Å in diameter and 35 Å deep, with radial symmetry creating a central pore 20 Å wide. Each protomer consists of 15 β-strands (labelled A to O from the N-terminus to the C-terminus) arranged in two large β-sheets in the form of a flattened β-barrel with jelly-roll topology. The β-sheets are held nearly perpendicular to the radial symmetry, creating two separate and distinct faces: the A face, which contains five α-helices, and the opposing B face, which contains five double metal-binding sites formed by the coordination of six localized protein residues. It is at these double metal-binding sites that CPHPC is bound. It is clear that these sites can readily accommodate two cadmium ions without any reorganization in spite of the substantial increase in atomic number over the natural calcium occupants of the sites. Five molecules of the bivalent CPHPC cross-link two SAP pentamers, which stack on a common fivefold axis to define a decamer 95 Å in diameter and 75 Å deep (see Fig. 1 ▶ *e*). The two proline carboxylate groups of each CPHPC molecule bind to the double metal-ion sites of subunits of opposing pentamers, giving an average O—Cd distance of 2.3 Å (Jesu Jaya Sudan & Sudandiradoss, 2012 ▶), with additional metal ligands provided by the side chains of Asp58, Asn59, Gln148, Glu136, Asp138 and the carbonyl of Gln137. The d-proline side chain fits into a pocket defined by the side chains of Tyr74, Tyr64 and Leu62 such that the pyrrolidine ring stacks against the ring of Tyr74 and forms van der Waals contacts with the other two side chains. The electron density for the six-carbon alkyl linker between the pyrrolidine rings shows a kinked rotamer at its centre, facilitating the approach of each head group to the metal site of the twofold axis-related subunit of the opposing SAP pentamer. Model building of the CPHPC ligand into the tenfold NCS averaged electron density of the lower resolution SAP–calcium map did not clearly establish whether the peptide bond preceding the proline ring was in the *cis* or the *trans* form, and in the deposited coordinates both *cis* and *trans* isomers were included for each ligand. In the high-resolution SAP–cadmium electron-density map we were able to fit two ligands as *trans* isomers in subunits *A* and *E*. In subunits *B* and *D* density for both *cis* and *trans* isomers was present, but was much stronger for the *cis* isomer (see Fig. 2 ▶). For subunit *C* it appears that the *cis* isomer is present, although the connecting density is weakest for this subunit. These assignments were based on the relative prominence of the electron density for the carbonyl O atom and the presence of alkyl-chain density sprouting from the carbonyl C atom in an OMIT map. Refinement was complicated by the five half-ligands residing in the asymmetric unit and critical density crossing the symmetry axis. Real-space refinement in *Coot* was complicated by the simultaneous presence of density for both isomers, and in subunits *B* and *D* the most satisfying fit was obtained with *cis* and *trans* isomers on a single molecule of CPHPC. However, in this space group the symmetry requires that the same isomer is present at each end of CPHPC, so in subunits *B* and *D* we presume that partial occupancy by both *cis*/*cis* and *trans*/*trans* isomers explains the density. In subunits *A* and *E* the carbonyl of CPHPC hydrogen-bonds to a water that is also a ligand to one of the cadmiums of the double-metal site of the opposing subunit (see Fig. 2 ▶). For bound *cis* isomers the carbonyls are directed away from this water and form hydrogen bonds to different water molecules. In addition to the five molecules of CPHPC and the cadmium ions, there are few direct protein–protein interactions in the decamer. The extended side chain of Arg77 hydrogen-bonding to Phe144 CO provides two good hydrogen bonds on each subunit pair and the alkyl chains of Arg77 and Lys143 are in close contact. At this resolution it is clear that the pentamers are displaced by rotation about their common fivefold axis by approximately 8°.

Good electron density was observed for portions of a biantennary oligosaccharide chain covalently attached to Asn32 of subunit *D* of the SAP pentamer wherever the chain interacted with the protein (see Fig. 1 ▶ *d*). Two terminal sialic acids and one covalently attached *N*-acetylglucosamine residue were well defined, but the density fades on moving away from these sites. Nevertheless, a plausible model was constructed, often building through water-molecule positions identified earlier in refinement. The terminus of one branch of the sugar chain hydrogen-bonds to Arg193 of the adjacent subunit (*E*) of the same pentamer, while the other branch forms a very similar interaction with Arg193 of a symmetry-related pentamer. In the other subunits these interaction regions are blocked by adjacent molecules in the lattice and electron density is only visible for the first protein-bound *N*-acetylglucosamine residue. While it is likely that the sugar chain is only partially ordered in this one location owing to packing, the fact that the same region of the protein is involved in both an intramolecular and an intermolecular interaction suggests that the intramolecular interaction may contribute to the normal stability of the isolated native pentamer.

### 3.2. *N*-Acetyl-d-proline complex   

Crystals of the SAP–*N*-acetyl-d-proline complex grew with a 45-fold molar excess of *N*-acetyl-d-proline in 60 m*M* Tris–HCl pH 8.0, 10 m*M* CaCl_2_, 84 m*M* NaCl, 20%(*v*/*v*) glycerol, 17%(*v*/*v*) PEG 550 MME. The crystals were monoclinic and X-ray diffraction data were collected from a single crystal to a resolution of 1.6 Å on beamline I03 at DLS (see Table 1 ▶). The molecular-replacement solution showed one SAP pentamer in the asymmetric unit and an estimated solvent content of 53%. Five d-proline ligands were observed bound to each SAP pentamer *via* the double calcium sites on each subunit. The puckered proline ring plane is parallel to that of Tyr74, but the penetration of the ligand into the pocket is not sufficiently deep to achieve stacking of all atoms. Rather, the proline C^γ^ and C^δ^ atoms interact with Tyr64, C^β^ and C^γ^ interact with Tyr74 and C^β^ interacts with Leu62. The proline NH is 4.2 Å away from Tyr74 OH. The peptide plane has been built as *cis*; this was a marginal decision based on the proximity of hydrogen-bond partners (the side-chain amide of Gln148 at 3.9 Å and the hydroxyls of Tyr64 at 4.6 Å and of Tyr74 at 5.5 Å). One molecule of glycerol was observed binding close to Pro29 on each subunit.

### 3.3. *N*-Acetyl-l-proline complex   

**Figure 3:**
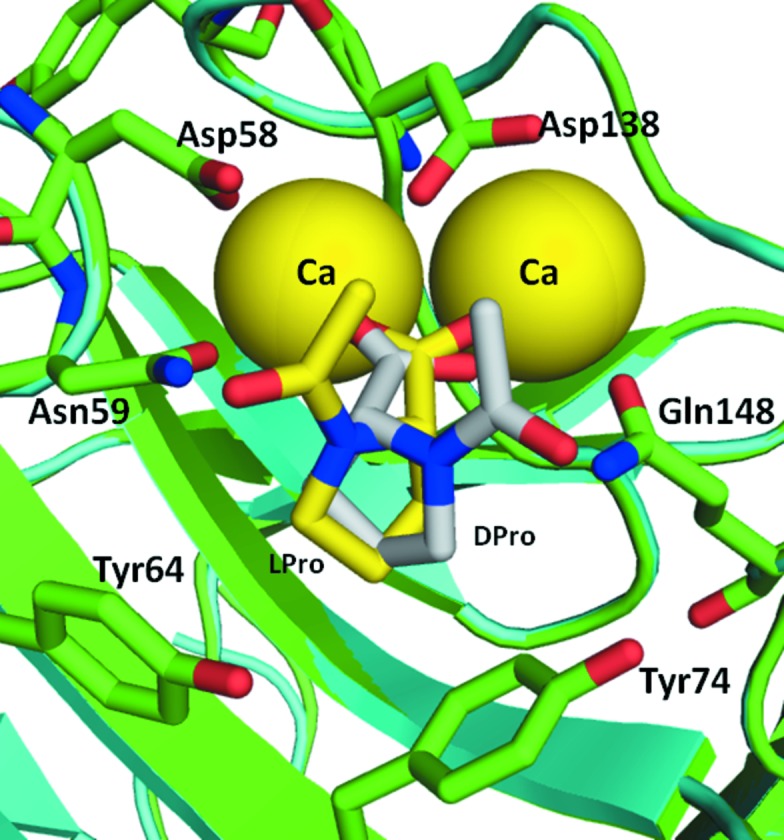
Cartoon of the superposition of SAP subunits with bound molecules of *N*-­acetyl-d-proline (yellow C atoms) and *N*-acetyl-l-proline (grey C atoms).

Crystals were obtained from drops containing a 50-fold molar excess of *N*-acetyl-l-proline with 60 m*M* Tris–HCl pH 8.0, 16%(*v*/*v*) PEG 550 MME, 10 m*M* CaCl_2_, 80 m*M* NaCl, 0.1%(*w*/*v*) NaN_3_ as the well solution. Data from a single monoclinic crystal were collected on station ID14-2 at the ESRF to 1.55 Å resolution (see Table 1 ▶). A single pentamer was found in the asymmetric unit by molecular replacement using *MOLREP*, giving a solvent content of 56%. l-Proline molecules were identified bound to each protein subunit. The positioning of the ligand is not unexpectedly altered compared with the d-proline isomer. The carboxylate interaction with the calcium ions is retained, but the pyrrolidine ring is rotated by approximately 90° owing to the reorganization at C^α^, and in response the position of Tyr74 changes by 0.46 Å. C^δ^ interacts with Tyr74, while C^β^ and C^γ^ interact with Tyr64. A superposition of the two structures is provided in Fig. 3 ▶.

### 3.4. CPHPC crystal structure   

**Figure 4:**
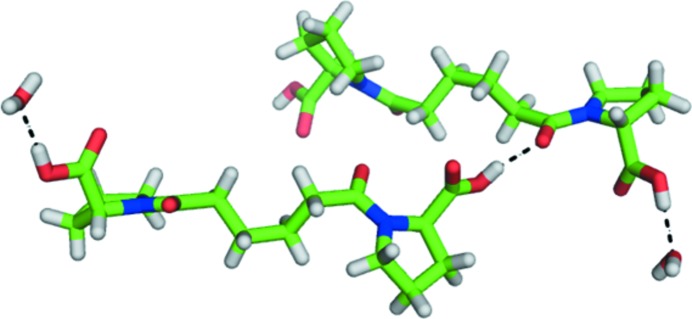
Two molecules of the lattice of crystalline CPHPC showing intermolecular hydrogen bonds of the *trans* isomer of the d-proline head group and the deviation of the alkyl chain from an extended form.

Crystal data for CPHPC: C_16_H_26_N_2_O_7_, *M*_r_ = 358.39, ortho­rhombic, space group *P*2_1_2_1_2_1_, *a* = 10.1602 (2), *b* = 10.4228 (2), *c* = 16.3042 (3) Å, *V* = 1726.58 (6) Å^3^, ρ_calc_ = 1.379 g cm ^−3^, μ = 0.108 mm^−1^, *Z* = 4, reflections collected 15 611, independent reflections 3932 (*R*_int =_ 0.0357), final *R* indices [*I* > 2σ(*I*)] *R*1 = 0.0370, *wR*2 = 0.0823, *R* indices (all data) *R*1 = 0.0420, *wR*2 = 0.0857. One partially extended molecule of CPHPC and one water molecule were present in the asymmetric unit. Both carboxylates were protonated and hydrogen-bonded either through a water molecule or directly to the amide-bond carbonyl of another molecule. Although a simple rotation about the C^α^–carboxylate bond would enable an intramolecular hydrogen bond with the amide-bond carbonyl, none were observed. The peptide bond preceding each pyrrolidine ring was in the *trans* conformation. Rather than a completely extended conformation, the central two C atoms of the linker adopted a *gauche* arrangement, tilting the proline-ring planes (see Fig. 4 ▶; CCDC deposition code 874670)^1^.

### 3.5. Calorimetry   

**Figure 5:**
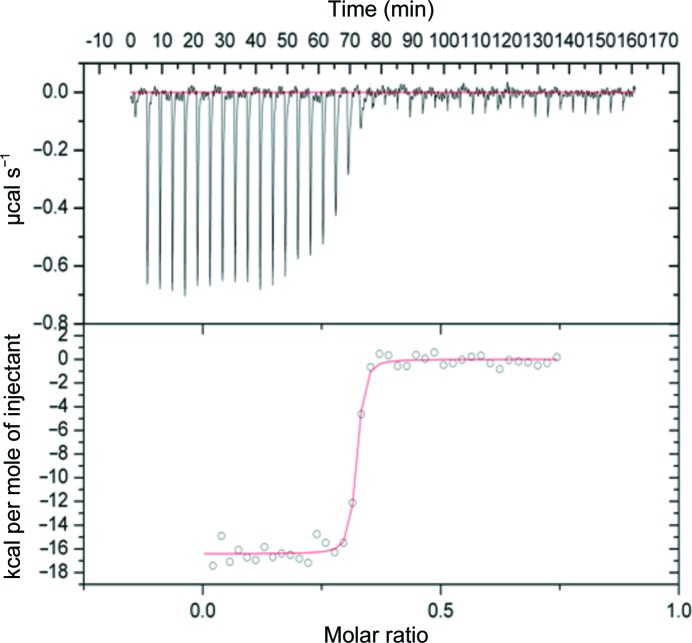
Isothermal titration calorimetry traces for the binding of CPHPC by SAP at 25°C and the best-fit curve (red) used to estimate *K*_d_.

Thermodynamic data from the binding of ligands is summarized in Table 2 ▶ and a typical isotherm is illustrated in Fig. 5 ▶. All calculations were based upon the SAP subunit concentration and the stoichiometry of ligand binding was not constrained. Nonetheless, this came very close to expectations, with approximately half a CPHPC molecule bound per subunit. The high affinity of the CPHPC interaction comes close to the limit of precise measurement by this titration procedure. The protein concentration required to give an appropriate heat change for each injection gave a high *c* value and few data points for accurate curve-fitting over the steep decline in the enthalpy curve (Wiseman *et al.*, 1989 ▶). A typical *K*_a_ value (SD) was 11.3 × 10^7^ (5.4 × 10^7^). The error in *K*_d_ for CPHPC is much larger than for the other ligands, but the results were reproducible in repeat experiments. Calculated *K*_d_ values show a 2000-fold increase in the apparent affinity for binding of half of the double-headed CPHPC compound by the SAP subunit: 8.8 n*M* compared with 18.6 µ*M* for *N*-acetyl-d-proline and 322 µ*M* for *N*-acetyl-l-proline. There appears to be a substantial entropic penalty associated with the binding of CPHPC that is broadly in accord with its larger number of freely rotatable bonds. This implies that substantially tighter binding might be achieved through linker rigidification, as shown by Ho *et al.* (2005 ▶).

### 3.6. HPLC of CPHPC   

**Figure 6:**
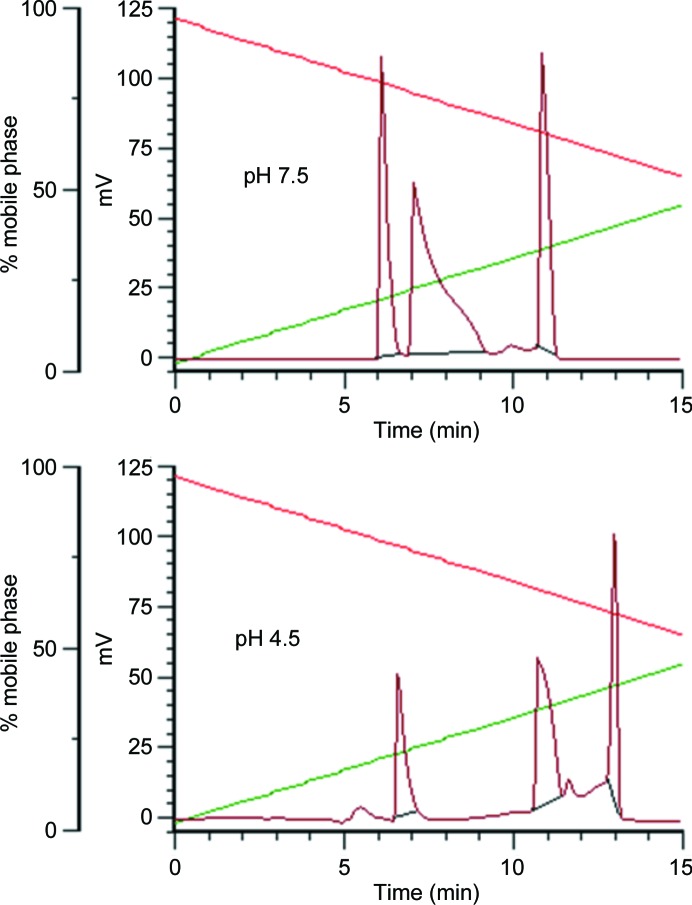
Elution profiles (UV absorption at 220 nm) of CPHPC from a reversed-phase column with acetonitrile gradients (acetonitrile, green line; aqueous, red line) at 4°C and pH values close to those employed in the crystallization of the protein complexes, showing three species corresponding to *cis*/*cis* (25%), *cis*/*trans* (45%) and *trans*/*trans* (30%) isomers at pH 4.5.

Elution of CPHPC from the reversed-phase column with a gentle acetonitrile gradient at pH 7.5 or 4.5 and 4°C showed three distinct peaks (see Fig. 6 ▶), all of which can regenerate the original chromatogram if collected and reapplied onto the column. These observations provide a close parallel to those reported for the antihypertensive drug captopril (Nishikawa *et al.*, 2004 ▶). This Cys-l-Pro inhibitor of angiotensin-converting enzyme oxidizes in the circulation, is excreted as a disulfide-linked palindrome analogous to CPHPC and is subject to *cis*/*trans* isomerization of the peptide bond preceding the proline. Reversed-phase HPLC of oxidized captopril under similar conditions to those reported here also generated three peaks that were attributed to *cis*/*cis*, *cis*/*trans* and *trans*/*trans* components eluting in that order. Based on this assignment, the *cis*/*trans* form of CPHPC is the dominant species, while the other two forms are present in approximately equal amounts (25–30%) at both pH values used in crystallization. At a lower pH of 3.0 the material redistributes towards a late-eluting peak that probably represents the *trans*/*trans* isomer.

## 4. Discussion   

Chemical cross-linking and gel-filtration experiments have previously confirmed that decameric SAP is stabilized by CPHPC in solution and is the species that forms and is cleared *in vivo* following administration of the drug (Kolstoe *et al.*, 2009 ▶). X-ray structure analysis of SAP in complex with CPHPC reveals the product of the cooperative high-affinity binding of five molecules of the bivalent drug by two SAP pentamers, cross-linking their B faces. In this interaction the apparent affinity of the head group rises by three orders of magnitude compared with isolated d-proline during the calorimetric titration of the palindromic compound. The two pentamers of SAP in the complex are out of register about their common fivefold axis by less than 10°, enabling the formation of ten good hydrogen bonds. Other investigations of ligand-cross-linked C-reactive protein (Pepys *et al.*, 2006 ▶) and SAP (Ho *et al.*, 2005 ▶) indicate that this rotation can vary considerably with linkers of different lengths. Optimal linker lengths are likely to be those that balance flexibity with enabling protein–protein interactions. The proximity of the pentamers required for such interactions is likely to be regulated by the shape of the opposing protein surfaces and the complementarity of charge and hydrogen-bonding capacity. The structure presented may be one of several possible low-energy rotamers.

While improved resolution of the structural analysis has been enabled by co-crystallization with cadmium ions, this has not entirely clarified the issue of any isomer preference for the CPHPC bound by SAP. In the conditions used here there are clearly preferred isomers for certain subunits and specific bonding interactions that might explain their presence. Isomer distribution does not appear to be a passive reflection of the solution composition during crystallization. HPLC shows that several isomeric forms of CPHPC can exist in solution and suggests that the mixed *cis*/*trans* isomer is the most abundant. However, this form is not consistent with the crystal symmetry. Crystal-packing effects and disorder might be invoked to explain the variability, but we are not able to provide a robust structural explanation for their influence on isomer preference at different positions. Subunits *A* and *E* show a clear preference for *trans* isomers and bonding interactions that stabilize them, but the partners in these interactions are still present in other subunits where *cis* isomers are bound. Although the proline ring of SAP is located in a pocket, there are no close interactions with protein side chains that might favour deconjugation of the peptide bond or pyramidalization of the proline N atom to promote isomerization within the binding site.

In solution, CPHPC shows a mixture of isomeric forms that can readily interconvert depending upon the conditions, and this behaviour is very similar to that of captopril and its oxidation product. NMR titrations of captopril showed a marked pH dependence of the isomer distribution, favouring the *trans* isomer at low pH (Rabenstein & Isab, 1982 ▶). This stability was attributed in part to the likely formation of an intramolecular hydrogen bond between the protonated carboxylate and the carbonyl O atom preceding the proline. It is of note that when the CPHPC carboxylate is coordinating the protein-bound cadmium ions and the pH dictates that it is ionized, there is no capacity for hydrogen bonding to the amide-bond carbonyl, removing this favourable interaction for stabilizing the *trans* isomer. However, this mode of hydrogen bonding is also not present in the all-*trans* crystalline CPHPC at low pH. Rather, in both cases a water molecule is the hydrogen-bond partner. Raising the temperature of the HPLC runs for captopril disulfide changes the isomer composition towards later-eluting peaks corresponding to the *trans*/*trans* isomer. At 37°C nearly all of the material elutes at this position as the energy barrier to isomerization is overcome and the most stable *trans* isomer is accessible. As CPHPC behaves in a very similar fashion, one might expect the *trans*/*trans* state to predominate *in vivo* at this temperature following drug administration.

In solution, the cross-linked SAP decamer may well be a flexible structure owing to the capacity for bond rotations in the ligand linker and sparse protein–protein interactions between SAP pentamers enabling their relative rotation. Binding of multiple cadmium ions at the pentamer interface probably damps this motion and provides a species that crystallizes rather well (Trakhanov *et al.*, 1998 ▶). From the structure of the complex, we see that cadmium ions outside the primary sites do not make interactions with the ligand and any influence that they may exert on the ligand conformation must be transmitted through the protein.

One can envisage a complex range of species emerging during the assembly of the decameric SAP–CPHPC complex. The most probable initial species are SAP pentamers with one or more CPHPC molecules attached through a single head group but with a distribution of *cis* and *trans* isomers of both head groups reflecting the equilibrium in solution. These species might preferentially interact with other constituents containing a complementary loading to form partially or fully loaded decamers. Less common collision complexes might result in pairs of SAP pentamers linked by a single or a small number of CPHPC molecules. In these cases the reduced degrees of freedom substantially raise the probability of binding further CPHPC molecules through both head groups in an escalating zipper-like manner. With a large excess of CPHPC one might expect all sites on an individual SAP pentamer to be filled with CPHPC through a single head group and for decamer formation to be inhibited. Indeed, we have previously reported that isolated human SAP runs as a single pentamer on size-exclusion chromatography in the presence of calcium and a 128-fold molar excess of CPHPC. Ho and coworkers described a crystal structure for such a non-cross-linked ligand–SAP complex (Ho *et al.*, 2005 ▶). They also described a structure for decameric SAP in which only three bivalent ligands are bound. Even if this crystal form was in part determined by crystal-packing effects, it does provide some evidence for stable, partially cross-linked SAP decamers implied by the discussion of assembly above. Similarly, *in vivo* infusion of CPHPC in humans initiates rapid depletion of plasma SAP when the molar ratio of CPHPC to SAP is just 1:1, indicating the formation of complexes composed of two SAP molecules cross-linked by just two CPHPC molecules.

The diastereoisomer of proline in CPHPC that was selected by high-throughput screening is not commonly found in biological systems. Nevertheless, we find that l-proline can be accommodated in the binding site of SAP, albeit with a tenfold reduction in affinity compared with the d isomer. Cooperative multipoint attachment of SAP to an l-proline-containing ligand could only promote this affinity with the involvement of an adjacent carboxylate. C-terminal proline residues that would fulfil this requirement are not common in known amyloid fibre proteins or their cleavage products. It seems most likely, therefore, that the high-throughput screen that identified the d-proline head group of CPHPC fortuitously located an atomic ensemble that mimics the amyloid motif recognized by SAP.

We have cited above various aspects of the SAP-bound form of CPHPC that might be varied to achieve tighter binding; variation of the linker length enables access to different pentamer rotations and locked *cis*-proline isomers are worthy of consideration since *cis*/*cis* isomers are bound in spite of their limited abundance. In a related way, Ho *et al.* (2005 ▶) reported a distinct chair form of the dioxane ring of the SAP-bound pyruvate acetal of glycerol that is not observed by NMR of the ligand in solution.

The efficacy of CPHPC *in vivo* in clearing circulating SAP is dramatic by virtue of the efficiency of hepatic recognition of the cross-linked SAP decamer. This ability to reduce plasma SAP concentrations with CPHPC is pivotal in the rapidly advancing use of CPHPC for the treatment of systemic amyloidosis (Gillmore *et al.*, 2010 ▶; Bodin *et al.*, 2010 ▶; http://www.clinicaltrials.gov/ct2/show/NCT01777243?term=amyloid+gsk&rank=2) and also offers exciting potential in Alzheimer’s disease (Kolstoe *et al.*, 2009 ▶). Furthermore, SAP binds avidly to DNA (Pepys & Butler, 1987 ▶) and may be responsible for the failure of DNA vaccination in humans compared with other species (Wang *et al.*, 2011 ▶). A clinical trial of SAP depletion in this context is currently in progress (M. B. Pepys, personal communication).

## Supplementary Material

Crystal structure: contains datablock(s) 06ac100. DOI: 10.1107/S1399004714013455/mn5064sup1.cif

PDB reference: serum amyloid P component, complex with d-proline, 4ayu

PDB reference: complex with l-proline, 4avs

PDB reference: complex with CPHPC and cadmium ions, 4avv

PDB reference: complex with CPHPC and calcium ions, 4avt

## Figures and Tables

**Table 1:** X-ray data-collection and refinement statistics for complexes of SAP with CPHPC, *N*-acetyl-D-proline and *N*-acetyl-L-proline Values in parentheses are for the highest resolution shell.

	*N*-Acetyl-L-proline	*N*-Acetyl-D-proline	CPHPC, calcium conditions	CPHPC, cadmium conditions
PDB code	4avs	4ayu	4avt	4avv
Space group	*P*2_1_	*P*2_1_	*P*4_3_2_1_2	*C*2
Unit-cell parameters (, )	*a* = 95.0, *b* = 70.0, *c* = 102.4, = 97.0	*a* = 94.4, *b* = 70.6, *c* = 99.1, = 96.9	*a* = 230.9, *b* = 230.9, *c* = 281.4, = = = 90	*a* = 154.4, *b* = 108.6, *c* = 120.3, = 138.5
Resolution range ()	30.41.4 (1.51.4)	31.21.5 (1.61.5)	24.93.2 (3.43.2)	80.61.6 (1.71.6)
No. of unique reflections	251592	198644	122013	170912
Multiplicity	3.6 (3.7)	7.0 (6.9)	3.9 (3.3)	3.7 (3.5)
Completeness (%)	96.4 (95.7)	96.5 (91.0)	98.0 (95.8)	99.2 (98.4)
*R*_p.i.m._ (%)	8.1 (26.2)	3.6 (38.5)	5.2 (38.9)	4.1 (13.2)
*I*/(*I*)	7.2 (3.5)	14.1 (2.6)	11.8 (2.0)	12.4 (5.0)
Wilson *B* factor (^2^)	8.47	14.7	81.8	14.7
Refinement				
Molecular-replacement model	1sac	1sac	1sac	1sac
No. of residues	1020	1020	2040	1020
No. of solvent molecules	1275	1362	0	1401
No. of other molecules	5 N7P, 10 Ca, 5 NAG	5 N8P, 10 Ca, 5 NAG, 5 GOL	10 GHE, 20 Ca, 10 NAG	5 GHE, 35 Cd, 8 ACT, 9 NAG, 2 MAN, 2 GAL, 3 SIA
R.m.s.d., bonds ()	0.010	0.007	0.015	0.010
R.m.s.d., angles ()	1.365	1.26	1.186	1.548
Ramachandran favoured (%)	98.3	98.3	95.9	97.3
Ramachandran outliers (%)	0.0	0.0	0.5	0.0
Average *B* factors (^2^)				
Protein	10.8	15.8	TLS used	16.6
Ligand	24.3	15.9	121.9	15.6
Solvent	27.5	31.8		29.8
*R* factor (%)	14.4	15.5	18.9	15.0
*R*_free_ (%)	17.0	16.8	19.7	17.5

**Table 2:** Thermodynamic parameters for isothermal titration calorimetry of SAP with CPHPC, *N*-acetyl-D-proline and *N*-acetyl-L-proline The error is expressed as the standard error of the mean for three experiments.

Ligand	*K*_d_ (*M*)	*H*_app_ (kcalmol^1^)	*T* *S* (kcalmol^1^)	*G* (kcalmol^1^)	*n* (stoichiometry)
*N*-Acetyl-L-proline	322.00 8.9	6.16 0.2	1.40 0.2	4.77 0.02	1.0
*N*-Acetyl-D-proline	18.60 2.2	3.44 0.6	2.69 0.5	6.12 0.4	0.9
CPHPC	0.0088 0.0026	14.87 0.8	3.83 0.7	11.04 0.2	0.4

## Footnotes

1 Supporting information has been deposited in the IUCr electronic archive (Reference: MN5064).
